# How Does Aging in Place Help Us Understand Loneliness During the COVID-19 Pandemic?

**DOI:** 10.1093/geroni/igab046.2209

**Published:** 2021-12-17

**Authors:** Judith Robertson Phillips, Cassandra Ford, Thomas Prohaska

## Abstract

Co-sponsored by the Disasters and Older Adults, Loneliness and Social Isolation, and Rural Aging Interest Groups, five presenters will highlight multiple circumstances regarding the intersection of social isolation or loneliness and the impact of COVID-19. Haverhals and colleagues interviewed veterans and their caregivers to identify the impact of changes in care delivery and social isolation as a result of the pandemic. Findings indicated differences in feelings of isolation among individuals living in their own home or assisted living facilities. Hua et al. examined whether individuals in long-term care communities were lonelier than individuals in the community during the pandemic using data from the NHATS COVID-19 module with higher levels of loneliness reported from individuals living in more restricted communities. Henning-Smith and colleagues explored differences in social activities among rural and urban participants in the COVID-19 Coping Study. Their study provides awareness into the ways rural and urban older adults stayed connected during the pandemic. Peterson et al. examined the effect of COVID-19 on care in Florida nursing homes and assisted living communities and on residents’ anxiety with higher levels of anxiety reported by residents in nursing homes. Using the Coping with Loneliness, Isolation and COVID19 Global Survey, O’Sullivan and colleagues utilized the lens of ‘place’ to examine factors associated with those experiencing loneliness and/or social isolation during the pandemic with insights from a public health perspective. Collectively, these presenters will provide evidence of the challenges associated with older adults’ social isolation and loneliness throughout the COVID-19 pandemic.

